# 

**Published:** 1873-05

**Authors:** 


☞ *The Editors are not responsible for the views of Contributors.*

